# Hierarchical multiple regression investigating factors associated with depressive symptoms in the middle-aged and elderly undergoing haemodialysis

**DOI:** 10.1186/s12889-023-15140-w

**Published:** 2023-02-03

**Authors:** Chou-Ping Chiou, Yu-Ling Bai, Liu-Yuan Lai, Hsiu-Chu Hsieh, Shin-Tsu Chang

**Affiliations:** 1 School of Nursing, I-Shou University, Kaohsiung, Taiwan; 2 Department of Nursing, Chung Hwa University of Medical Technology, Tainan, Taiwan; 3 Department of Nursing, Fooyin University Hospital, Pingtung, Taiwan; 4 Department of Nursing, Kaohsiung Armed Forces General Hospital, Kaohsiung, Taiwan; 5 Department of Physical Medicine and Rehabilitation, Kaohsiung Veterans General Hospital, Kaohsiung, Taiwan; 6 Department of Physical Medicine and Rehabilitation, School of Medicine, Tri-Service General Hospital, National Defense Medical Center, Taipei, Taiwan

**Keywords:** Depressive symptom, Haemodialysis, Middle-aged and elderly, Social support, Rehabilitation, Health care professional

## Abstract

**Background:**

Depressive moods are commonly seen in patients who receive haemodialysis. This can cause a lack of compliance in their treatment procedures and increase the rate of hospitalization. This study aimed to investigate the relationship between social support and degree of depression in middle-aged and elderly patients undergoing haemodialysis and the predictors of depressive symptoms.

**Methods:**

A cross-sectional correlational study was designed with a structured questionnaire survey. Patients over 40 years of age were included from five haemodialysis centres. Measures embraced a demographic and clinical characteristics questionnaire, the Centre for Epidemiologic Studies Depression Scale, and the Personal Resource Questionnaire 2000. Statistical analysis was performed using hierarchical multiple regression analysis.

**Results:**

A total of 179 patients over 40 years of age were included from five haemodialysis centres in the analysis. The mean CES-D score was 19.0(12.3); the majority of participants (60.3*%*) had a CES-D score ≥ 15, indicating likely depressive status. The mean PRQ2000 score was 75.7(15.9). The proportional mean of the PRQ2000 was 72.11*%*, indicating moderate social support for participants in this study. Data disclosed that marital status, number of comorbidities, exercise behaviour, and social support could significantly predict depressive symptoms; total explanatory variance was 31.3%.

**Conclusion:**

Health care professionals should identify those at high risk of depressive symptoms when they provide care to the middle-aged and elderly patients undergoing haemodialysis. These findings may lead to greater insights into the nursing and rehabilitative care of patients treated by chronic maintenance haemodialysis.

## Background

Patients with end-stage renal disease (ESRD) receiving haemodialysis (HD) always suffer lifestyle changes due to the physical and psychological impact of their disease and treatment. The development of feelings of depression is one major issue of concern [1–3] for these individuals. Depressive moods are not infrequent in patients receiving HD. This often leads to their failure to comply with treatment actions [8]. Symptoms of depression can affect patients’ ability or desire to continue with their lifestyle and treatment, worsen their quality of life [9], increase frequent hospitalization [3], and raises the risk of death [10].

Previous studies have shown that gender, educational level [1], serum creatinine concentration [6], and duration of HD [7], are associated with depressive symptoms in patients receiving HD. The illiterate patients had more depressive symptoms [4, 5]. Depression symptoms in older HD patients were notably higher than the age-matched controls [4]. Elderly patients undergoing HD had significantly higher levels of depression than same-age patients without HD [11]. A previous study mentioned that age over 60 years, marital status, and low economic status are risk factors for depressive symptoms [12].

An annual report of the Taiwan Renal Registry Data System on kidney disease shows that the number of patients older than 40 years of age continues to increase each year; therefore, this associated problem will continue to grow [13]. Statistical data from multiple studies in Taiwan has shown that the majority of patients with ESRD undergoing HD are middle-aged or elderly; those over 40 years of age account for 80.7–95.6% of the total group of patients undergoing HD [10, 14]. A few studies have evaluated the prevalence of depression among middle-aged and elderly patients undergoing HD; the reported prevalence rates of depression in patients undergoing HD older than 60 years of age is 45.9–50% [15, 16]. However, depressive moods are often overlooked and underestimated in older patients receiving HD [10]. The root cause crisis is the negative aspects of physiological changes. We ascertained that illness and psychological health issues in middle-aged and elderly patients with ESRD are of critical importance and cannot be ignored.

Demographic data, for instance, age, marital status, and economic status, are risk factors of depressive symptoms in patients undergoing HD. By contrast, Cengić and Resić [17] have shown that sociodemographic information, such as gender, marital status, and duration of HD, do not notably affect depressive symptoms. Additionally, Palmieri et al. [4] found that the age of the patients and duration of HD are not preponderant factors for the occurrence of depressive symptoms. Social support is important for patients undergoing HD [8]; worse family cohesion is associated with depressive symptoms [18]. Further, depressive symptoms have a significant influence on adherence by mediating the effects of family and healthcare provider support [19]. Moreover, perceived social support to treatment maintenance in patients undergoing HD is associated with lower depressive symptoms [15]. Even though there are many reports about the background factors on depressive symptoms in patients undergoing HD, more information is needed to clarify and confirm the results in different populations. Further, whether these factors can predict depressive symptoms in patients undergoing HD continues to require further investigation.

This study aimed at investigating depression levels and relative influencing factors (including demographical variables, clinical variables, and social support) in middle-aged and elderly patients with ESRD undergoing HD in Taiwan. We hypothesized that the recognition of factors relating to depressive symptoms can orient health care professionals (HCPs) in clinical practice. This will help them assess psychological condition and provide the necessary care and support.

## Methods

### Sample and setting

The researchers collected questionnaire data for this cross-sectional study. Based on a power analysis using a moderate effect size and probability level of 0.05 and 0.80 power, a sample size of at least 145 participants was deemed adequate [20]. Participants receiving HD were recruited from five HD centres in southern Taiwan, which are categorized in the same tier of medical teaching hospitals and were accounted as indistinguishable consensus among the five due to annual accreditation by way of identical criteria. Individuals over 40 and 65 years old were defined as middle-aged and elderly, respectively. The exercise was instructed by members of rehabilitation team in each hospital. This study followed the acceptance criteria of patients undergoing long-term HD as those who had experienced HD for over three months. This was defined by the National Health Insurance Administration (NHIA), Ministry of Health and Welfare (MOHW) of Taiwan to recruit suitable participants for the study. We recruited individuals who met the criteria and agreed with our research purpose. Inclusion criteria were ≥40 years of age; alert and oriented; able to understand and speak Mandarin; and have undergone HD for at least three months. Exclusion criteria were comorbid diagnosis of cancer and dementia; those who needed to adapt to treatment by controlling their eating, drinking, and medicine; and those who were affected by an unstable patient lifestyle and HD disequilibrium syndrome. The participant recruiting process was executed by doctors and researchers. The survey was conducted in private spaces where participants were recruited. For those participants with low literacy, we provided assistance, i.e., researchers read questions and recorded responses throughout the data collection period. Participants took 20–40 mins to complete their questionnaire.

### Instruments

The research instruments comprised a demographic and clinical characteristics questionnaire and two scales. The demographic characteristics included age, gender, education level, marital status, source of income, living situation, and exercise behaviour. Clinical characteristics included number of comorbidities, duration of HD, and type of vascular access, and most recent pre-HD plasma creatinine results.

Social support was measured using PRQ2000, which is a self-administered questionnaire consisting of 15 positively-posed questions. It uses a 7-point Likert scale; responses ranged from 1 (strongly disagree) to 7 (strongly agree). Mental health measures were used to examine the validity of PRQ2000. The construct validity for the PRQ2000 substantiated that social support is related to mental health constructs in the anticipated direction and strength. In this study, an alpha coefficient of 0.95 was obtained. The concept of social support was defined as five dimensions: attachment/ intimacy, social integration, nurturance, reassurance of worth, and availability of assistance. Total scores for this questionnaire ranged from 15 to 105, with higher scores representing higher levels of social support. To derive the scores of each measurement, a proportional mean was calculated as the mean score divided by the maximum score, and then multiplied by 100. Validity and reliability for the PRQ2000 test data for adults and older adults has shown an internal consistency alpha reliability ranging from 0.87 to 0.93 [21]. The Chinese version of this questionnaire was in a study involving elderly patients with diabetes [22].

Depressive symptoms were assessed using the Chinese version of the Centre for Epidemiologic Studies Depression Scale (CES-D Scale), which is a popular short self-report scale with concurrent validity by clinical interview schedule and substantial evidence of construct validity [23]. This scale is a 20-question with 4-point Likert-type scale, from 0 to 3. Total scores extend from 0 to 60; higher scores represent more depressive symptoms [24]. In Taiwan, the cut-off point to indicate depressive symptoms is a CES-D score of 15 or higher. The CES-D Scale had an internal consistency reliability with a Cronbach’s α of 0.76 [14]. The internal consistency reliability was 0.93 in this study, as assessed using Cronbach’s α.

### Ethical considerations

Before commencement of this study, the objectives and methods were explained to participants. Questionnaires were anonymous and information was confidential. Participants had the option of terminating the questionnaire and withdrawing from the study at any time and their participation did not affect the rights of patients to receive treatment. Data were collected only after receiving a completed consent form.

### Data analysis

Data were analysed using IBM Statistical Package for Social Sciences (SPSS, version 22.0; IBM Corporation, Armonk, NY: USA). Data examination was performed before analysis to determine the presence of outliers; data distribution was assessed for normality to ensure that the data conformed to basic statistical hypotheses. Demographical descriptive data and clinical characteristics of the subjects were analysed and presented as descriptive statistics including means, standard deviations (SDs), and percentages. For inferential statistics, hierarchical multiple regression analysis was conducted to determine factors contributing to depression status. Hierarchical multiple regression modelling was conducted with depressive symptoms as the dependent variable to examine depression level and relative influencing factors. A three-step process was followed. In the first regression model, the sociodemographic factors were entered as independent variables. The second regression model included clinical variables as additional independent variables. Measures of support were added into the final regression model.

## Results

### Demographical descriptive data and clinical characteristics

Initially, 190 people met the criteria to serve as participants; however, 11 refused to participate. Ultimately there were 179 valid questionnaires. An expediency sample of 179 participants receiving HD was enrolled for analysis. As shown in Table 1, the mean age was 63.8(10.6) years and the mean education level was 7.0(4.5) years. In this study, 131 participants (73.2*%*) listed another person as their source of income, 126 (70.4*%*) had a spouse, and only 15 (8.4*%*) were living alone. In total, 145 (81*%*) participants received arteriovenous fistula and the duration of HD ranged from 3 to 225 months (mean = 72.1).

**Table 1:** Demographic and clinical variables of subjects (N = 179)

Variables	n	%	Mean (SD)
Age (year)			63.8(10.6)
Gender			
Female	93	52	
Male	86	48	
Marital status			
With spouse	126	70.4	
No spouse	53	29.6	
Source of income			
Others	131	73.2	
Individual	48	26.8	
Living alone			
Yes	15	8.4	
No	164	91.6	
Education (year)			7.0(4.5)
Duration of HD (months)			72.1(58.1)
Type of vascular access			
AVF	145	81	
AVG	34	19	
Creatinine(mg/dl)			9.9(2.1)
Number of comorbidities			2.2(0.9)
0	49	27.4	
1	65	36.3	
2	44	24.6	
≥3	21	11.7	
Exercise behaviour(times/week)			3.2(2.0)
0	62	34.6	
1	18	10.1	
2	19	10.6	
3	27	15.1	
4	9	5	
≥5	44	24.6	
Social support			75.7(15.9)
Depressive symptoms			19.0(12.3)
0–14	71	39.7	
≥15	108	60.3	

### Depressive symptoms and social support scores for HD patients

Table 1 showed the scores of depressive symptoms and social support in the study population. The mean CES-D score was 19.0(12.3) (range, 0–49); the majority of participants (60.3*%*) had a CES-D score ≥ 15, indicating likely depressive status. The mean PRQ2000 score was 75.7(15.9) (range, 29–105). The proportional mean of the PRQ2000 was 72.11*%*, indicating moderate social support for participants in this study.

### Predictors of depressive symptoms for HD patients

All characteristics in our study were divided into three sets. These were individually analysed using hierarchical multiple regression analysis. Model 1 included six independent demographical characteristics: age, gender, marital status, education level, source of income, and living situation. Model 2 included five independent clinical characteristics: number of comorbidities, creatinine level, duration of HD, exercise behaviour, and vascular access. Model 3 included social support.

As shown in Table 2, the predictor demographical descriptive characteristics in model 1 could notably clarify degree of depression (*F* = 3.63, *p* < 0.01); however, the only variable to reach significance was marital status (β = −0.27, *t* = − 3.53, *p* = 0.001). The degree of depression was lower for those with a spouse than for those without (model explanatory variance = 11.2*%*). Age, gender, educational level, source of income, living situation, creatinine level, duration of HD, and vascular access did not significantly predict the degree of depression in patients undergoing HD. After controlling for demographical characteristics and introducing clinical characteristics in model 2, the results showed a statistically significant correlation to clarify the degree of depression with 19.8*%* variability (*F* = 3.76, *p* < 0.001). The explanatory variance increased by 8.6*%*, with number of comorbidities and exercise behaviour both reaching significant levels. The predictive power of exercise behaviour was higher; beta = − 0.21 (*t* = − 2.73, *p* < 0.01), indicating less exercise was significantly associated with greater depressive symptoms. The beta for number of comorbidities was 0.19 (*t* = 2.45, *p* < 0.05), indicating that having more comorbidities was significantly related to greater depressive symptoms. After incorporating social support in model 3, including all independent characteristics, the total explanatory variance rose to 26*%* (*F* = 6.31, *p* < 0.001), specifying that the additive social support increased explanatory variance by 11.5*%*. The explanatory variance of social support for depressive symptoms was statistically significant (beta = − 0.36, *t* = − 5.64, *p* < 0.001), presenting that less social support was notably associated with greater depressive symptoms.

**Table 2:** Hierarchical regression analysis for variables predicting depressive symptoms (N = 179)

Predictor variable	Model 1β	Model 2β	Model 3β
Age	0.10	0.13	0.14
Gender	0.01	−0.09	−0.13
Marital status (No spouse: 0; With spouse: 1)	−0.27**	−0.21**	−0.15*
Education level	0.01	0.04	0.07
Source of income (Others: 0; Individual: 1)	−0.13	−0.14	−0.11
Living alone (No: 0; Yes: 1)	0.00	0.04	0.03
Number of comorbidities		0.19*	0.17**
Creatinine		0.15	0.13
Duration of HD		−0.00	−0.00
Exercise behaviour		−0.21**	−0.14*
Vascular access (AVG: 0; AVF: 1)		−0.05	−0.07
Social support			−0.36***
R^2^	0.11	0.20	0.31
Adjusted R^2^	0.08	0.15	0.26
R^2^ change	0.11	0.09	0.12
F	3.63**	3.76***	6.31***

*P < 0.05; ** P < 0.01; *** P < 0.001

HD: haemodialysis; AVF: arteriovenous fistula; AVG: arteriovenous graft

Goodness of fit testing was performed for the regression analysis using a residual analysis of standardized residual z-scores. There were five, six, and eight sample observations, respectively, for the 179 participants in the three models that fell outside the ± 1.96 SD (outside of the 95*%* confidence interval). A number of 95*%* of sample observations were within a reasonable range, showing that the goodness of fit of these three models and data were good. Additionally, this showed that prediction using regression models with these variables was appropriate.

## Discussion

Our data obtained from middle-aged and elderly patients undergoing HD showed that individuals had moderate social support and low personal financial conditions, and a high proportion of patients had depressive symptoms and lack of exercise. The significant predictors of depressive symptoms analysed from the hierarchical multiple regression included multiple comorbidities, lack of exercise, moderate social support, and no spouse.

The average number of comorbidities was 2.2 (0.9), which is slightly inconsistent with the results of Liu et al. [15]. They reported that 194 elderly patients in Taiwan undergoing HD had an average of 1.64 comorbid conditions. This inconsistency might be only commonly seen in comorbidities listed in ESRD in our study, such as cardiovascular disease, hypertension, and autoimmune disease. Moreover, our results showed that 34.6*%* of participants did not exercise, even when the rehabilitation team was involved. This was similar to results from Li et al. [25], which revealed that 26.7*%* of 187 patients who underwent HD were inactive. Factors influencing exercise might include not realising the benefits of exercise, a lack of desire to exercise due to feeling unwell, not having time or equipment to perform exercise, or a lack of instruction from a rehabilitation team member. Dziubek et al. [26] have found that the application of regular physical exercise can improve psychological status and lowers depressive symptoms in patients undergoing HD. However, the training program originally used for patients with hypercholesterolemia [27], dementia [28], chronic heart failure [29], and myofascial pain dysfunction syndrome [30], might be very inappropriate if used on HD patients.

Social support for participants was moderate (72.1*%*) in this study. This result was similar to the results from a previous study involving 202 Iranian adult patients who received HD [31]. Up to two thirds of the participants had a spouse and over 90% were living with family or in an institution; therefore, they were able to obtain social support from family and friends. Nevertheless, our CES-D results indicated that 60.3*%* of participants exhibited depression. This differs from a previous study that reported that Leinau et al. [32], who depicted 27*%* of 109 patients undergoing HD aged 45 years or older were depressed, based on US scoring with the Patient Health Questionnaire. However, both studies show that patients who receiving HD tended to have psychological problems that required the time and support of HCPs.

Hierarchical multiple regression analysis found that middle-aged and elderly patients undergoing HD who had a spouse had fewer depressive symptoms than those without a spouse. This finding was similar to that of Park et al. [12], who have reported that unmarried status is a predictor for depressive symptoms. This may be related to stability and close relationships within patient families. Our study verified that number of comorbidities is a predictor for depressive symptoms. Comorbidities have a negative impact on depressive symptoms; therefore, the influence of comorbidities on health and depression should be elucidated so that patient health conditions can be understood and the appropriate care is provided. This study did not examine the relationship between different comorbidities and depression, which could be explored in more depth in future studies, such as diabetes and thyroid dysfunction. Additionally, this study found that exercise behaviour can significantly predict depressive symptoms. This indicated that exercise decreases depressive symptoms. Therefore, HCPs should aid patients in selecting the appropriate exercise and encourage activity to improve health.

This study found that social support can predict the level of depression in these patients. Social support increased explanatory power by 11.5% in the hierarchical multiple regression model, indicating that this is an important predictor. This is consistent with the findings of Khalil and Abed [2]. They have reported that perceived social support is negatively associated with depressive symptoms in patients undergoing HD. Patients’ resilience to depressive symptoms can be increased by providing social support through care, emotional help, and open discussion [33]. Health care providers could assist patients with support sources so that they can face the negative impact of disease. They can provide patients with realistic and helpful support. For example, teaching patients to think positively, helping them to follow a healthy diet, and providing medical and non-medical subsidies for financially unstable patients.

Age, gender, educational level, source of income, living situation, creatinine level, duration of HD, and vascular access had no significant influence on levels of depression. Alencar et al. [9] have also confirmed depressive symptoms could not be predicted by age and gender. Although Park et al. [12] conducted linear logistic regression on patients < 60 and ≥ 60 years of age has shown that age > 60 years is a risk factor for depressive symptoms, the influence of age was not prominent in this study. Therefore, its negative impact must be researched further in subsequent studies. According to this study, gender is not associated with depression symptoms, but menopause may also cause depression, therefore, deeper investigation into the causes of depression in men and women is necessary. A previous study showed that depressive symptoms are correlated positively with lower educational level [4]. However, our study was unable to support any correlation between education level and depressive symptoms. This may be due to the fact that 48.2*%* of patients in the study of Semaan et al. [4] had completed either high school or a university degree, while the average years in education for participants in our study was 7.01 ± 4.51. The impact of educational level on depressive symptoms must be researched further in subsequent studies. Further, living alone did not predict depressive symptoms in our study. This may be due to the low number of participants living alone in our cohort (*n* = 15, 8.4*%*) or due to different participant groups. Prospective studies that including a larger sample of those living alone can be used to investigate its potential effect on depressive symptoms.

A previous study of 47 patients showed that depressive symptoms are positively correlated with high serum creatinine concentrations [6]. However, creatinine levels did not predict depressive symptoms in this study. This difference might be due to inconsistent representation in this cohort (179 vs. 47). An additional previous study has shown that depressive symptom in patients undergoing HD is significantly negatively correlated with duration of HD [1]; however, other studies have shown no significant correlation. This indicates that the duration of HD is unable to significantly predict depressive symptoms, like the conclusions of Cengić and Resić [17] and ours. Leinau et al. [32] found that depression is not correlated with type of vascular access, which was verified in our study.

## Limitations and future direction

One limitation of the study is that participants were not randomly selected. In addition, the sample was enrolled from five dialysis centres in southern Taiwan. The generalizability of this finding to other patients undergoing HD in other geographical areas cannot be assured.

This study found that middle-aged and elderly patients who underwent HD have a high prevalence of depression. Additionally, these results showed some important factors that influence depressive symptoms. Middle-aged and elderly patients undergoing HD require HCPs to recognise their psychological problems; therefore, depressive symptoms should be regularly checked. HCPs should assess factors related to patients’ tendency for depression, such as social support, marital status, and comorbidities. HCPs should use the time to interact with patients who are scheduled to undergo HD for three to four hours and three times per week. Additionally, HCPs should verbally encourage the patients to provide positive thinking and reduce negative emotions. This study showed that exercise and social support are significant factors that reduce depressive symptoms and should be considered in the care of patients undergoing HD. Regular exercise or habitual physical activity is a fundamental part of living [34–37]. HCPs, particularly team members of rehabilitation, should help patients discover a suitable mode of exercise and provide guidance for home exercise. Under permitting circumstances, HD centres may be used as a location for activities; they can offer exercise apparatus and habitual dealings for patients. This can enhance opportunities for social interaction that can aid patients to adjust to the bearing of their disease and improve their depressive symptoms. Our results showed that 34.6% of participants in this study did not exercise; therefore, future studies should investigate whether decreased exercise behaviour is related to disease process or progression. Moreover, this research offers an insight for HCPs in these patients, for example information on their experience depressive symptoms.

## Conclusions and recommendations

This study revealed moderate social support for middle-aged and elderly Taiwanese patients. Additionally, a large proportion of these patients had depressive symptoms. The predictors of these symptoms included marital status, comorbidities, exercise, and social support. As a result, HCPs should consider these factors during the care of these patients. Future studies may provide a more in-depth understanding of the psychosocial dimension of these patients, the role of rehabilitation, or differences in depressive symptoms and their influencing factors among patients of different age groups.

## Data Availability

Transcripts that support the findings of this study are not publicly available but anonymised datasets used and/or analyzed during the current study are available from the corresponding author upon reasonable request.
